# Chronic expanding hematoma with a significantly high fluorodeoxyglucose uptake on ^18^F-fluorodeoxyglucose positron emission tomography, mimicking a malignant soft tissue tumor: a case report

**DOI:** 10.1186/1752-1947-8-349

**Published:** 2014-10-21

**Authors:** Yusuke Nishida, Eisuke Kobayashi, Daisuke Kubota, Nokitaka Setsu, Koichi Ogura, Yoshikazu Tanzawa, Fumihiko Nakatani, Yoshiharu Kato, Hirokazu Chuman, Akira Kawai

**Affiliations:** 1 Department of Musculoskeletal Oncology, National Cancer Center Hospital, 5-1-1 Tsukiji, Chuo-ku 104-0045, Tokyo, Japan; 2 Department of Orthopaedic Surgery, Tokyo Women’s Medical University, 8-1 Kawada-cho, Shinjuku-ku 162-8666, Tokyo, Japan

**Keywords:** Chronic expanding hematoma, FDG-PET, Soft tissue sarcoma

## Abstract

**Introduction:**

Chronic expanding hematoma is a rare persistent hematoma that can sometimes be misdiagnosed as a malignant tumor due to its clinical and radiological features.

**Case presentation:**

A 42-year-old Japanese man with a large mass in his leg, suggestive of malignancy, presented to our hospital. He had been aware of the leg swelling for the last eight years. A magnetic resonance imaging scan demonstrated a large mass with two components. One was a large, well-defined cystic mass (13×9cm) showing high intensity on T1- and T2-weighted images, and the other was a solid mass (3.5×2.5cm, adjacent to the large mass) with high intensity on T1-weighted images. Two-[^18^F]fluoro-2 deoxy-D glucose positron emission tomography images revealed increased uptake with a maximum standardized uptake value of 15.8 in the solid mass. As these findings were considered suggestive of hematoma associated with a malignant lesion, an open biopsy was performed. A pathological examination demonstrated a hematoma with xanthogranuloma, and no malignant cells were evident. Therefore, we resected the tumor including both components, and the histological diagnosis was chronic expanding hematoma. Clinical diagnosis based on 2-[^18^F]fluoro-2 deoxy-D glucose uptake is sometimes limited by the fact that 2-[^18^F]fluoro-2 deoxy-D glucose is taken up by not only malignant tumor cells but also macrophages and tissues with granulation or inflammation.

**Conclusions:**

Significantly increased standardized uptake value in the peripheral rim of the lesion on 2-[^18^F]fluoro-2 deoxy-D glucose positron emission tomography imaging, mimicking a soft tissue sarcoma, should be recognized as a potential diagnostic pitfall in cases of chronic expanding hematoma.

## Introduction

Chronic expanding hematoma (CEH) is a rare lesion that was first defined by Reid *et al.* in 1980 [1]. CEH is characterized by its persistence and increasing size over a period of more than a month after the initial hemorrhage, whereas most hematomas in skeletal muscle may arise with or without any identifiable trauma and gradually expand over days to weeks, followed by a decrease in size or disappearance within a few months. Therefore, CEH can be misdiagnosed as a malignant soft tissue sarcoma because of its clinical characteristics such as a large size and slowly progressive enlargement [1,2]. Two-[^18^F]fluoro-2 deoxy-D glucose positron emission tomography (FDG-PET) is an evolving diagnostic modality used for tumor detection, staging, therapeutic monitoring and follow-up evaluations for the treatment and management of various malignant tumors [3,4].

Here we present a case of CEH in the lower extremity. The lesion exhibited significantly increased FDG uptake on FDG-PET, mimicking the characteristics of a soft tissue sarcoma accompanied by hematoma.

## Case presentation

**Figure 1:**
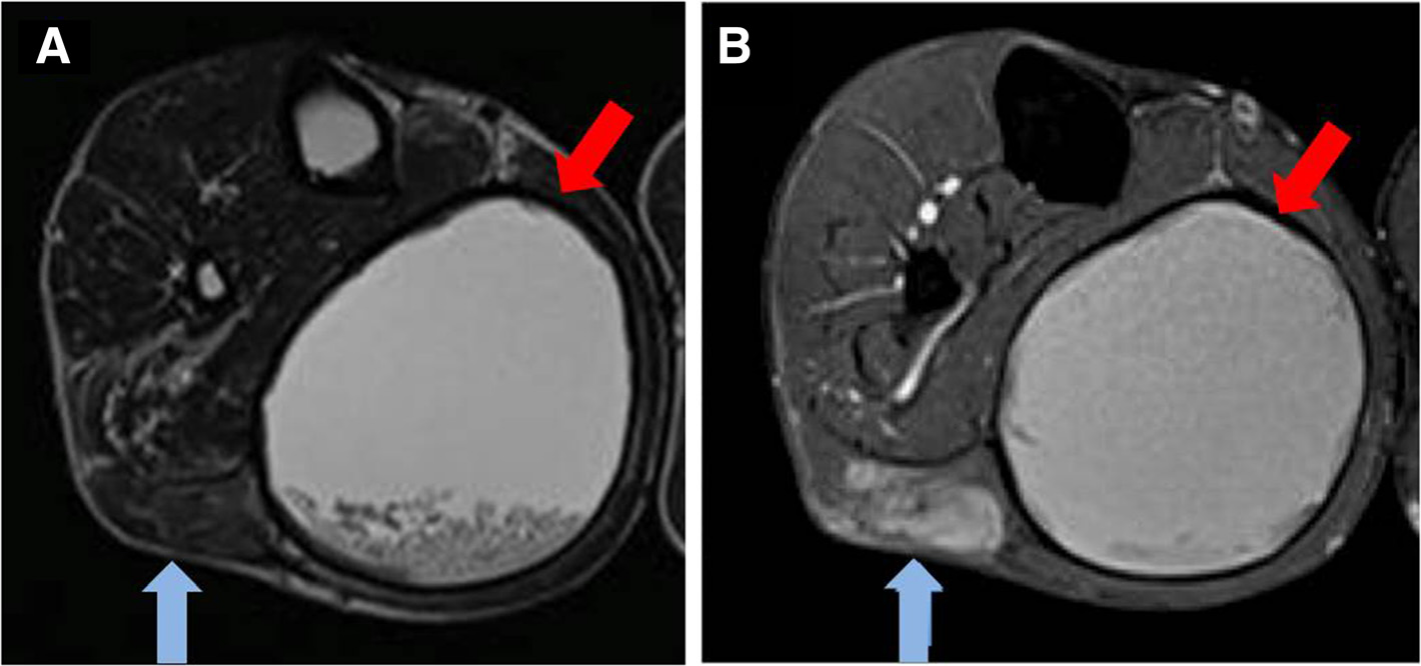
**Magnetic resonance imaging scan.** Axial magnetic resonance imaging (T2, Gd-enhanced) views show a well-defined cystic mass (red arrow) with debris in the right medial calf **(A)**, and a Gd-enhanced solid lesion in the medial gastrocnemius (blue arrow) **(B)**.

**Figure 2:**
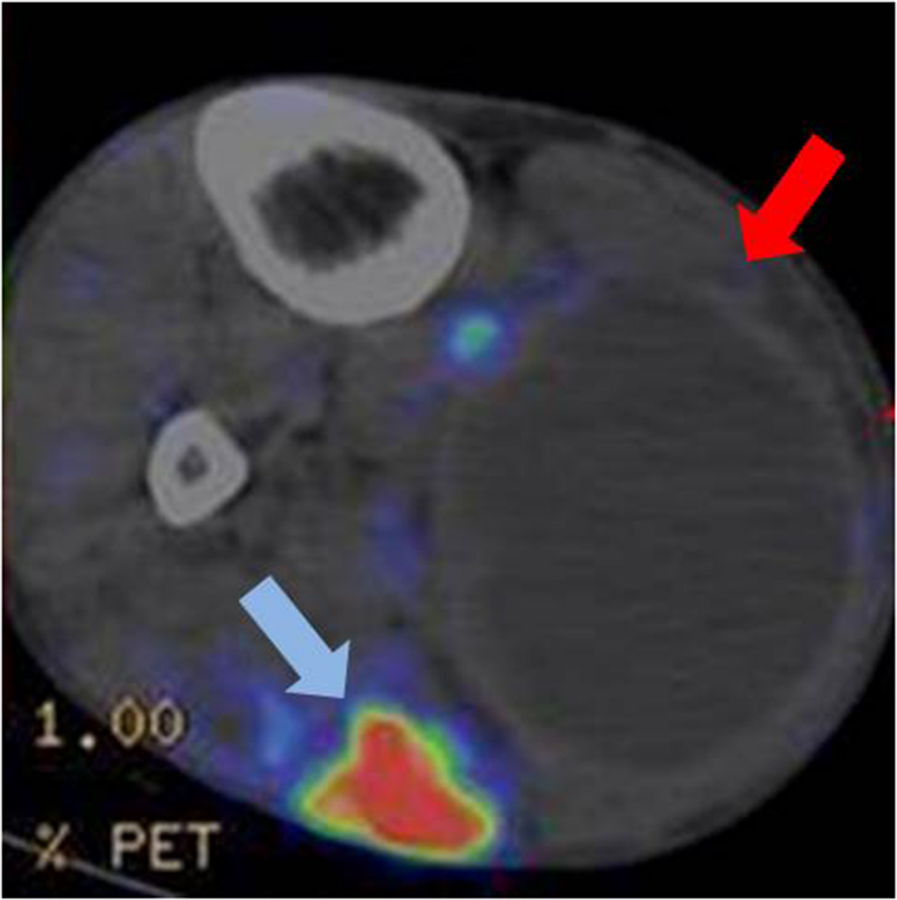
**Two-[**^**18**^**F]fluoro-2 deoxy-D glucose positron emission tomography image.** Axial fused 2-[^18^F]fluoro-2 deoxy-D glucose positron emission tomography image shows abnormal 2-[^18^F]fluoro-2 deoxy-D uptake (maximum standardized uptake value: 15.8) in the solid lesion (blue arrow) adjacent to the cystic lesion (red arrow).

**Figure 3:**
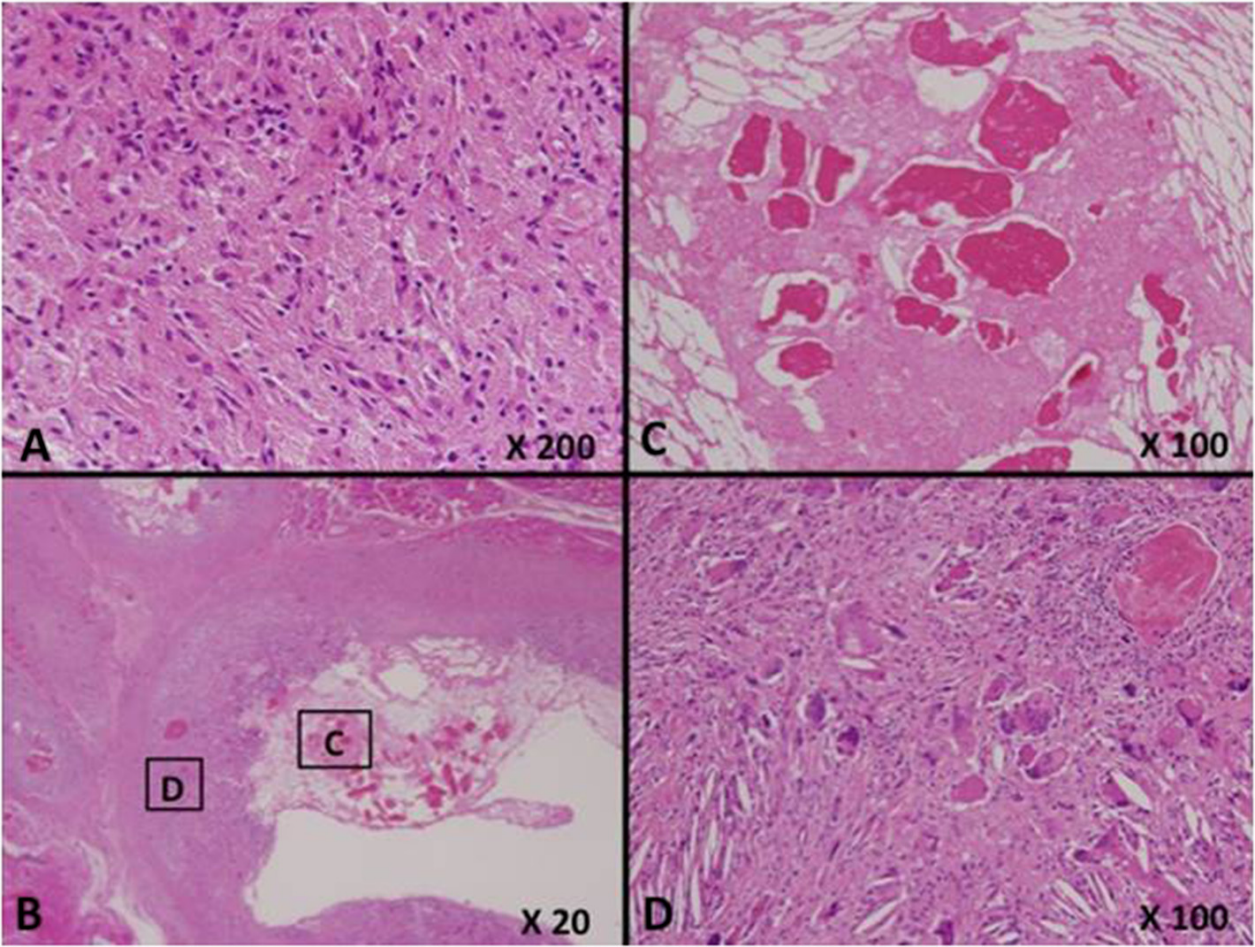
**Histopathological features. (A)** The biopsy specimen shows a mixture of hematoma and xanthogranuloma, with no malignant cells. **(B-D)** A hematoma accompanied by accumulation of fibrin, cholesterin crystals, and histiocytes.

A 42-year-old Japanese man was referred to our hospital because of pain in his right leg. He had first become aware of pain in his leg during a rugby game eight years previously. Although the pain had subsequently improved, his leg had remained swollen for several years, without having suffered any obvious trauma or received any anticoagulant therapy. Before referral to our hospital, he had developed renewed pain in his leg for one month. Upon physical examination, a large hard mass was palpable in his right leg. A magnetic resonance imaging (MRI) scan showed a 13×9-cm cystic mass with high signal intensity on T1- and T2-weighted images between the soleus and lateral gastrocnemius, and a 3.5×2.5-cm solid lesion with high signal intensity on T1-weighted images in the medial gastrocnemius. This solid lesion adjacent to the cystic mass was enhanced on Gd-enhanced T1-weighted images (Figure 1A, B). FDG-PET images revealed increased uptake with a maximum standardized uptake value (SUV) of 15.8 in the solid lesion (Figure 2). As these clinical findings and diagnostic images were considered suggestive of hematoma associated with a malignant lesion, an open biopsy of both components was performed. A pathological examination revealed that the lesions consisted of a mixture of hematoma and xanthogranuloma with no malignant cells (Figure 3A). Therefore, complete resection of the masses was performed based on a diagnosis of CEH after obtaining informed consent. During the operation, no active hemorrhaging or a source of bleeding was identified. The resected specimen was a dark brown, partially organized hematoma surrounded by fibrous tissues. A postoperative pathological examination yielded a diagnosis of hematoma accompanied by accumulation of fibrin, cholesterin crystals, and histiocytes in the cystic region (Figure 3B-D). As of eight months after surgery, our patient is in good health with no sign of recurrence.

## Discussion

Reid *et al.* first used the term ‘chronic expanding hematoma’ for hematomas that persisted and increased in size for more than a month after the initial hemorrhage event [1]. Such lesions often simulated neoplastic growth. On the other hand, it has been reported previously that some soft tissue sarcomas, including synovial sarcoma, epithelioid sarcoma, extraskeletal Ewing sarcoma, and malignant fibrous histiocytoma, sometimes exhibit the radiological features of a hematoma. Such clinical and radiological features often make the accurate diagnosis of pure hematoma difficult. The mechanisms that determine the formation of CEH are not well understood. Labadie and Glover theorized that breakdown of leukocytes, hemoglobin, platelets, and fibrin results in an inflammatory process that effectively damages the capillaries of the capsule, increasing the permeability of the vascular wall and causing bleeding from dilated microvessels underneath the fibrous capsule [5].

Various kinds of imaging modalities have been utilized for diagnosis of CEH. FDG-PET imaging is increasingly being used in clinical oncology because it enables functional imaging of various tumors. Generally, high-grade sarcomas and aggressive benign lesions such as desmoid-type fibromatosis and giant cell tumor of tendon sheath tend to have higher SUVs than other benign lesions [6]. However, it is also known that tumor diagnosis based on FDG-PET imaging is limited by the fact that FDG is taken up by not only tumor cells but also macrophages, granulation and inflammation tissue [7]. High uptake of FDG has been observed in many kinds of inflammatory lesions, including chronic osteomyelitis, tuberculosis, and rheumatoid arthritis. A previous study has also indicated that macrophages and immature granulation tissue containing fibroblasts contribute to the increase uptake of FDG in tumors [8]. Knight *et al.* has also reported that active granulomatous processes such as tuberculosis accumulate FDG and cause false positivity during PET evaluation of malignancy [9]. In the present case, the solid lesion containing xanthogranulation showed increased FDG uptake. To date, the FDG-PET imaging features of CEH have not been clearly established. A few past reports have indicated that the peripheral portion of a CEH arising from the thorax or pelvic cavity tends to take up FDG within a SUV_max_ range of 3.1 to 5.5 (Table 1). This pattern is also evident in soft tissue sarcomas with a tendency for central necrosis. To the best of our knowledge, such a significantly high FDG uptake (SUV_max_ was 15.9) in a CEH arising from the extremity has not been reported previously. In this case, despite increased FDG uptake in the solid lesion at the peripheral rim, the final histological diagnosis was CEH with no malignancy. Therefore, a significantly increased SUV in the peripheral rim of CEH in FDG-PET images should be recognized as a potential diagnostic pitfall mimicking a soft tissue sarcoma with hematoma formation.

**Table 1:** Fluorodeoxyglucose positron emission tomography imaging characteristics of chronic expanding hematoma

**Authors**	**Sex/Age (years)**	**Location**	**Size (cm)**	**Treatment**	**SUV**_**max**_
Hamada *et al.*[10]	M/65	Ilium	8	Resection	3.1
Kwon *et al.*[11]	F/67	Hemithorax	ND	Resection	3.7
Takahama *et al.*[12]	M/77	Chest wall	4.5	Open puncture	5.5
Tokue *et al.*[13]	M/77	Intrapericardial	9	Resection	3.5
Present case	M/42	Leg	13	Resection	15.8

FDG, 2-[18F]fluoro-2 deoxy-D glucose; PET, positron emission tomography; SUV_max_, maximum standardized uptake value; ND, no data.

Although the treatment of CEH is still a matter of debate, most authors have recommended surgical resection. Aspiration or drainage of the material may not remove the clot contents fully, and undoubtedly will not remove the fibrous wall that might retain fluid. Additionally, aspiration of the fluid or incomplete excision could lead either to an unconfirmed diagnosis or to recurrence [14]. Hanagiri *et al.* reported that the surgical procedure should be complete resection, because incomplete resection might result in massive bleeding from a hypervascular subcapsular lesion [15]. Therefore, it seems that complete surgical resection, including the pseudocapsule, is the best treatment option for CEH.

## Conclusions

In summary, we have presented a case of CEH showing significantly increased FDG accumulation, which could have had the potential for misdiagnosis as a soft tissue sarcoma.

## Consent

Written informed consent for publication of this case report and any accompanying images was obtained from the patient. A copy of the written consent form is available for review by the Editor-in-Chief of this journal.

## Competing interests

The authors declare that they have no competing interests.

## Authors’ contributions

YN and EK prepared and drafted the manuscript and performed the literature research. EK and AK edited the manuscript and performed the literature research. DK and NS edited the manuscript and performed the literature research. YT and FN edited the manuscript. HC and YK edited the manuscript. EK and KO were the operating surgeons, and were involved in the preparation of the manuscript. All authors read and approved the final manuscript.
